# Variability of Creatine Metabolism Genes in Children with Autism Spectrum Disorder

**DOI:** 10.3390/ijms18081665

**Published:** 2017-07-31

**Authors:** Jessie M. Cameron, Valeriy Levandovskiy, Wendy Roberts, Evdokia Anagnostou, Stephen Scherer, Alvin Loh, Andreas Schulze

**Affiliations:** 1 Genetics and Genome Biology, Peter Gilgan Center for Research and Learning, Toronto, ON M5G 0A4, Canada; jessie.cameron@sickkids.ca (J.M.C.); Valeriy.Levandovskiy@sickkids.ca (V.L.); Stephen.Scherer@sickkids.ca (S.S.); 2 Department of Paediatrics, University of Toronto, Toronto, ON M5S 1A1, Canada; wendy.roberts@sickkids.ca (W.R.); Evdokia.Anagnostou@sickkids.ca (E.A.); Alvin.Loh@SurreyPlace.on.ca (A.L.); 3 Holland Bloorview Kids Rehabilitation Hospital, 150 Kigour Rd, Toronto, ON M4G 1R8, Canada; 4 The Centre for Applied Genomics and Genetics and Genome Biology, the Hospital for Sick Children, Toronto, ON M5G 1X8, Canada; 5 McLaughlin Centre and Department of Molecular Genetics, 686 Bay Street, 13th Floor, Peter Gilgan Center for Research and Learning, Toronto, ON M5G 0A4, Canada; 6 Surrey Place Center, 2 Surrey Place, Toronto, ON M5S 2C2, Canada; 7 Department of Biochemistry, University of Toronto, Toronto, ON M5S 1A8, Canada

**Keywords:** autism spectrum disorder, creatine deficiency syndrome, *glycine amidinotransferase*, *guanidinoacetate methyltransferase*, *solute carrier family 6 member 8*, genetic variability

## Abstract

Creatine deficiency syndrome (CDS) comprises three separate enzyme deficiencies with overlapping clinical presentations: arginine:glycine amidinotransferase (*GATM* gene, glycine amidinotransferase), guanidinoacetate methyltransferase (*GAMT* gene), and creatine transporter deficiency (*SLC6A8* gene, solute carrier family 6 member 8). CDS presents with developmental delays/regression, intellectual disability, speech and language impairment, autistic behaviour, epileptic seizures, treatment-refractory epilepsy, and extrapyramidal movement disorders; symptoms that are also evident in children with autism. The objective of the study was to test the hypothesis that genetic variability in creatine metabolism genes is associated with autism. We sequenced *GATM*, *GAMT* and *SLC6A8* genes in 166 patients with autism (coding sequence, introns and adjacent untranslated regions). A total of 29, 16 and 25 variants were identified in each gene, respectively. Four variants were novel in *GATM*, and 5 in *SLC6A8* (not present in the 1000 Genomes, Exome Sequencing Project (ESP) or Exome Aggregation Consortium (ExAC) databases). A single variant in each gene was identified as non-synonymous, and computationally predicted to be potentially damaging. Nine variants in *GATM* were shown to have a lower minor allele frequency (MAF) in the autism population than in the 1000 Genomes database, specifically in the East Asian population (Fisher’s exact test). Two variants also had lower MAFs in the European population. In summary, there were no apparent associations of variants in *GAMT* and *SLC6A8* genes with autism. The data implying there could be a lower association of some specific *GATM* gene variants with autism is an observation that would need to be corroborated in a larger group of autism patients, and with sub-populations of Asian ethnicities. Overall, our findings suggest that the genetic variability of creatine synthesis/transport is unlikely to play a part in the pathogenesis of autism spectrum disorder (ASD) in children.

## 1. Introduction

Autism spectrum disorder (ASD) can be present in children with inborn errors of metabolism, the latter having a prevalence of 1 in every 800 live births [1]. Less than 5% of ASD cases can be attributed to routinely-screened-for inborn errors of metabolism [2,3]. Creatine deficiency syndrome (CDS) is an inherited metabolic disorder that is not included in routine newborn screening panels or in standard ASD diagnostic work up. The presence of autistic symptoms in patients with CDS suggests that genes in the creatine metabolic pathway may play a part in the neurobiology of ASD and may represent a treatable cause of ASD [4]. We recently ascertained the prevalence of CDS in children with non-syndromic autism in a large, prospective, multicentre study by screening for creatine metabolites in urine and sequencing *GAMT*, *GATM* and *SLC6A8* genes (glycine amidinotransferase, guanidinoacetate methyltransferase and solute carrier family 6 member 8) for pathogenic mutations (443 children with ASD). The estimated prevalence of CDS was less than 7 in 1000 and there was no obvious correlation between pathogenic CDS gene mutations and ASD, Therefore, the chances of a child with ASD having a CDS were very low [5].

In the study presented here, we investigate the association of autism with non-CDS disease causing variants in the three creatine metabolism genes. Whereas non-synonymous nucleotide changes resulting in amino acid changes are likely to result in pathogenic mutations, synonymous changes that do not alter the amino acid are often attributable to polymorphisms. It has been shown that single nucleotide polymorphisms (SNPs) can explain up to 40% of the variance in liability to ASD [6,7]; thus common genetic polymorphisms can be a major risk factor for autism.

The clinical presentation of CDS is characterized by developmental delays and cognitive decline, intellectual disability, impaired speech and language, autistic behaviour, epileptic seizure activity, treatment-refractory epilepsy, and extrapyramidal movement disorders [8,9,10,11]. Significant behavioural, social and communication challenges seen in ASD can be caused by neurodevelopmental disabilities of complex aetiology. The symptoms in patients with CDS and those with ASD can overlap.

In patients with CDS, the impaired synthesis or transport of creatine leads to depletion of creatine/phosphocreatine in the brain. The creatine–phosphocreatine system plays an essential role in cellular energy homeostasis in the central nervous system. This system acts as a temporal and spatial energy buffer, energy transducer and general regulator of cellular energetics [12]. Creatine has been shown to act as a neuroprotective agent [13]; it protects rat hippocampal neurons against glutamate and amyloid beta peptide toxicity [14]; it seems to function as a potent natural survival and neuroprotective factor for γ-aminobutyric acid (GABA)-ergic neurons in a model for Huntington disease [15] and of dopaminergic neurons in a model for Parkinson’s disease [16]. In two studies, creatine supplementation was convincingly shown to improve concentration [17], as well as short-term memory and learning [18] in healthy human subjects. Due to these protective functions of creatine the deficiency of creatine could play a part in the neurobiology of ASD.

CDS includes three separate deficiencies: arginine:glycine amidinotransferase (AGAT), guanidinoacetate methyltransferase (GAMT), and creatine transporter (CrT) [4,8,19]. AGAT and GAMT deficiencies are autosomal recessive inherited biosynthesis defects of creatine. The AGAT gene (NCBI Gene ID 2628, official nomenclature: *GATM*) located on chromosome 15q15.3 is 16.8 Kb in size and contains nine exons, which encode 424 amino acids. The *GAMT* gene (Gene ID 2593) located on chromosome 19p13.3 is 4.46 Kb in size and has six exons encoding 237 amino acids. Dysfunction of the CrT leads to impaired intra-cellular creatine uptake and has X-linked inheritance. The CrT gene (Gene ID 6535, *SLC6A8*) located on Xq28 spans 8.4 Kb and consists of 13 exons encoding 635 amino acids [4]. To date, there are 6, 29, and 88 pathogenic mutations described throughout the *GATM*, *GAMT*, and *SLC6A8* genes, respectively [20]. The common denominator of all these diseases is the virtually complete absence of creatine and phosphocreatine in the brain that can be assessed with in vivo magnetic resonance spectroscopy (MRS). The diagnosis can be made in spot urine determining the concentration of creatine and guanidinoacetate normalized for creatinine, in 24-h urine measuring the excretion of creatine, guanidinoacetate, and creatinine, and by measuring the concentration of these metabolites in blood [4,21,22]. Among CDS, GAMT deficiency, while presenting as a spectrum from mild to severe, has the most severe phenotype that includes severe intellectual deficits. Treatment-refractory epilepsy and extrapyramidal movement disorders are exclusively seen in GAMT patients [4,8,10,23,24]. The few AGAT-deficient patients described so far seem to have a less severe presentation with developmental delays and impaired communication and social contact. Mild seizures have been reported in only some of the cases [9,25,26]. Males with X-linked CrT deficiency present with impairments of speech and language and may have a seizure disorder. They usually show behavioural abnormalities and develop intellectual disability. While most of the female carriers have a normal appearance, some may have mild learning difficulties. Treatment with supplementation of creatine can fully (AGAT) or partly (GAMT) replenish brain creatine and attenuate the clinical course in AGAT and GAMT-deficient patients. In contrast, creatine supplementation has no effect on brain creatine and the clinical course in male CrT patients [4]. There is an even gender distribution in GAMT [10] and likely in AGAT deficiencies, whereas predominantly males are affected with the CrT defect. The latter is an X-linked condition and female carriers are mostly asymptomatic [8].

Because of the overlap of symptoms between CDS and ASD and because of the potential role of the creatine-phosphocreatine homeostasis in autism, we hypothesize that comparing the ASD population to the general population may reveal an altered frequency of genetic variants in the three CDS genes (*GATM*, *GAMT* and *SLC6A8*) that are associated with autism.

## 2. Results

A total of 166 subjects enrolled in the study, with 71 from the prospective group enrolled in Toronto and 95 from the Genome Canada study sample. Of these, 32 were females, and 134 were males. Ethnicities were described as European “EUR”, Asian “ASN”, African “AFR” or Admixed/Unknown, “MIX/UNK” (Supplementary Table S1).

### 2.1. Novel/Rare Variants Observed in CDS Genes in ASD Patients

**Figure 1:**
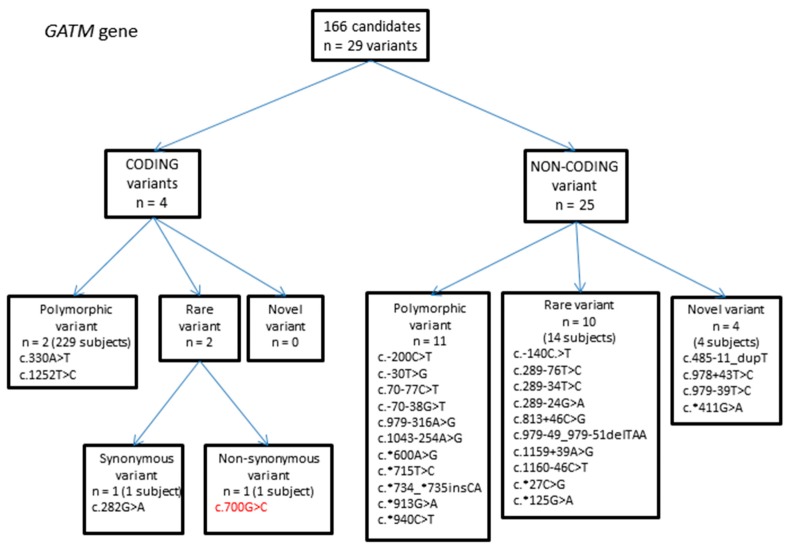
Flowchart summarizing variants identified in the glycine amidinotransferase (*GATM*) gene.

**Figure 2:**
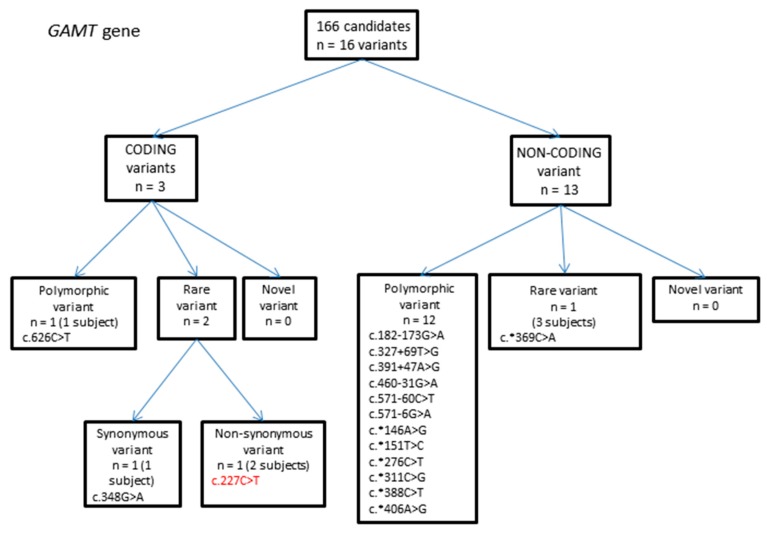
Flowchart summarizing variants identified in the guanidinoacetate methyltransferase (*GAMT*) gene.

**Figure 3:**
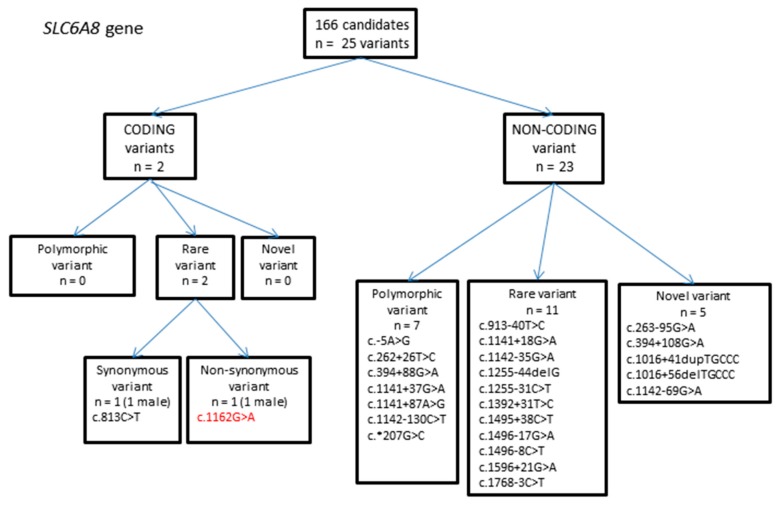
Flowchart summarizing variants identified in the solute carrier family 6 member 8 (*SLC6A8*) gene.

Sequence data was compared with NCBI reference genes, and genetic variations were compared to three variation databases: the 1000 Genomes phase 3 dataset, the National Heart, Lung and Blood Institute Grand Opportunity Exome Sequencing Project (NHLBI GO ESP) and the Exome Aggregation Consortium (ExAC). A number of variants were identified, some of which are novel (not in the variant databases) or rare (present at allele frequencies less than 1% or 0.01) (Table 1, Table 2 and Table 3, Figure 1, Figure 2 and Figure 3).

#### 2.1.1. *GATM* Gene Variants

A total of 29 variants were identified, of which four were present in coding regions (Figure 1, Table 1). Of these, two were rare variants: one was synonymous and one was non-synonymous. Synonymous variants are presumed to not have any potential pathogenicity, unless they are likely to affect splicing. The non-synonymous variant (c.700G > C, p.Asp234His) was not present in 1000 Genomes or ESP, but was present in the ExAC database at a minor allele frequency of 0.00001651 (2/121104 chromosomes, mixed ethnic population) (Table 1). The variant was identified as heterozygous in one individual in the ASD population, resulting in a minor allele frequency (MAF) of 0.003 (1/332 chromosomes). Computational software (SIFT [27], PolyPhen-2 [28] and MutationTaster [29]) all predict this variant to be damaging, and therefore pathogenic if homozygous (Table 1). Further functional testing would be needed to confirm the true pathogenicity of this variant.

**Figure 4:**
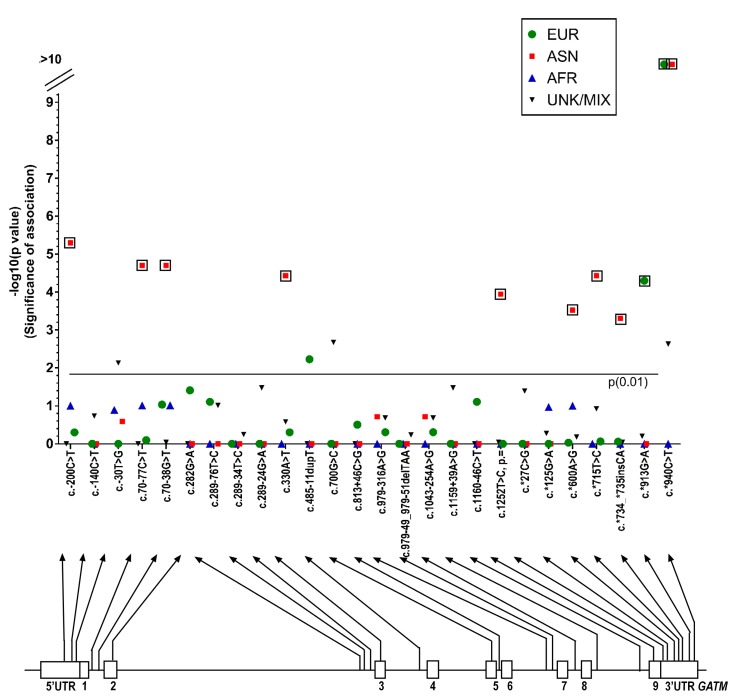
Manhattan plot showing the *p*-values from the Fisher’s exact test (significance of association, plotted as -log (*p*)) for each SNP sequenced on the glycine amidinotransferase (*GATM*) gene. *p*-values are shown for European, East Asian, African, and Unknown/Admixed populations. The nominal statistical threshold for *p* = 0.01 is shown, and *p*-values that are still significant after running the Benjamin–Hochburg procedure are indicated with boxes. The location of each SNP is shown on the *GATM* gene below the x-axis. EUR: European; ASN: East Asian; AFR: African; and UNK/MIX: unknown/admixed populations.

There were several differences seen in the MAFs calculated for *GATM* variants identified in the ASD patient group compared to those from ethnic populations in 1000 Genomes database (European, East Asian, African and Mixed/Unknown) (Supplementary Table S2). Figure 4 shows the *p*-values from the Fisher’s exact test (plotted as -log (*p*)) and the variants identified. Fifteen of these differences were initially found to be significant (Fisher’s exact test, *p* = 0.01), and after running the Benjamini–Hochberg procedure, 11 of these values remained above the threshold of significance. Nine of these values represented a significant difference between the allele frequencies seen in our autism population and the East Asian population of the 1000 Genomes database: SNPs rs7164139 (c. − 200C > T, *p* = 0.000005), rs12437887 (c.70 − 77C > T, *p* = 0.00002), rs12437840 (c.70 − 38G > T, *p* = 0.00002), rs1288775 (c.330A > T, *p* = 0.000037), rs1145086(c.1252T > C, *p* = 0.000114), rs1049503 (c.*600A > G, *p* = 0.0003), rs1049508 (c.*715T > C, *p* = 0.000037), rs35410548 (c.*734_*735insCA, *p* = 0.000499) and rs1049518 (c.*940C > T, *p* = 0), Figure 4). All nine variants had a significantly lower MAF than the 1000 Genomes East Asian population database (Supplementary Table S2). It is possible that some of these SNPs could be inherited as a single haplotype (adjacent SNPs that are inherited together). To further investigate these nine variants, we compared the data from our Asian population with the 1000 Genomes South Asian population (Table 4). This data suggested that none of the variants were significantly different.

Two variants in the European cohort show significant variation from the 1000 Genomes European database population: rs17618637 (c.*913G > A, *p* = 0.00005) and rs1049518 (c.*940C > T, *p* = 0) both in the 3′UTR (untranslated region) of the *GATM* gene. Both had lower MAFs than in the database (Supplementary Table S2).

#### 2.1.2. *GAMT* Gene Variants

A total of 16 variants were identified, of which three were in coding regions (Figure 2, Table 2). Two of these were rare variants, and one of these was non-synonymous. This variant (c.227C > T), observed in a heterozygous state in two individuals, had an MAF of 0.000616 in the ESP database (8/12984 chromosomes) and 0.000411 in the ExAC database (29/70554 chromosomes) (Table 2). In our ASD population, the variant was observed in 2/332 chromosomes, resulting in an MAF of 0.006. SIFT, PolyPhen-2 and MutationTaster all predicted the variant to be damaging (Table 2).

**Figure 5:**
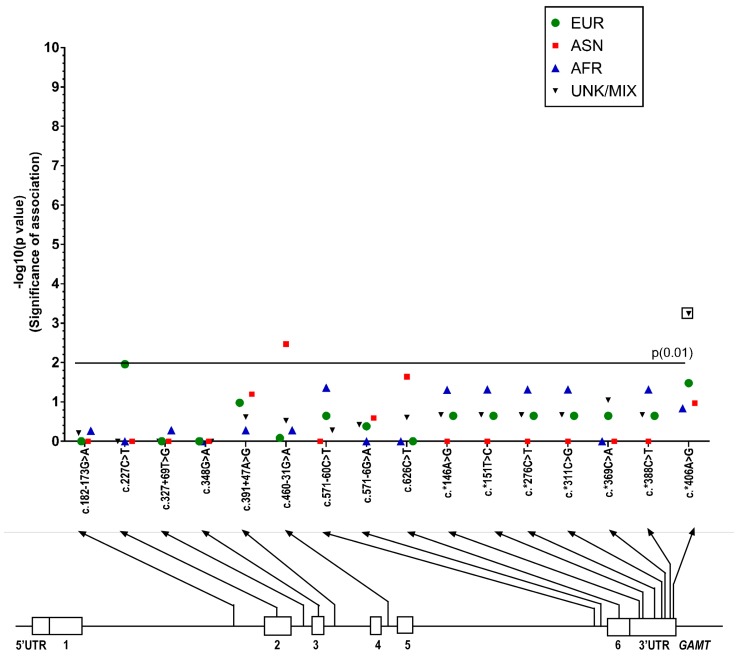
Manhattan plot showing the *p*-values from Fisher’s exact test (significance of association, plotted as -log (*p*)) for each SNP sequenced on the guanidinoacetate methyltransferase (*GAMT*) gene. *p*-values are shown for European, East Asian, African, and Unknown/Admixed populations. The nominal statistical threshold for *p* = 0.01 is shown, and *p*-values that are still significant after running the Benjamin–Hochburg procedure are indicated with boxes. The location of each SNP is shown on the *GAMT* gene below the x-axis. EUR: European; ASN: East Asian; AFR: African; UNK/MIX: Unknown/Admixed populations.

There were some differences seen in the MAFs calculated for *GAMT* variants identified in the ASD patient group compared to those from ethnic populations in the 1000 Genomes database (Supplementary Table S3). Figure 5 shows the *p*-values from Fisher’s exact test (plotted as −log (*p*)) and the variants identified. Only three of these differences were significant (Fisher’s exact test, *p* = 0.01), and after running the Benjamini–Hochberg procedure only one of these remained significant (r266813).

#### 2.1.3. *SLC6A8* Gene Variants

In total 25 variants were identified, of which two were in coding regions (Figure 3, Table 3). Of these, one resulted in a rare, non-synonymous coding change in exon 8: c.1162G > A. This was identified as hemizygous in a male individual, which would imply the variant would cause disease if found to be pathogenic, since the *SLC6A8* gene is on the X chromosome. The MAF was 0.005 (1/198 alleles), and allele frequencies for the 1000 Genomes, ESP and ExAC databases were 0.0003 (1/3775 alleles), 0.0002886 (3/10394 alleles) and 0.00007051 (1/14182 alleles) respectively. SIFT, PolyPhen-2 and MutationTaster all suggest the variant is damaging, and if so, the variant could potentially be disease causing. However, the variant is listed in the Leiden Open Variation Database v2 [30] and is shown by one study to be benign. The variant was identified in a patient with mental retardation, but fibroblasts from the patient were confirmed to have normal creatine uptake. The variant was seen in 2/1900 patients with mental retardation [31]. Two more rare variants were also identified as non-pathogenic by other studies: c.813C > T, a synonymous variant in exon 5 [32], and c.1142 − 35G > A in intron 7, as well as several of the non-coding polymorphic variants [33].

**Figure 6:**
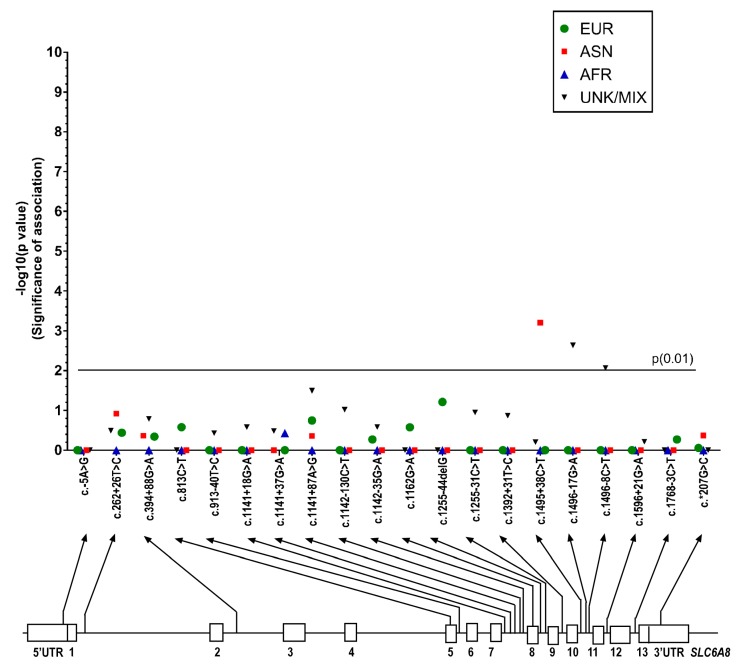
Manhattan plot showing the *p*-values from Fisher’s exact test (significance of association, plotted as -log (p)) for each SNP sequenced on the solute carrier family 6 member 8 (*SLC6A8*) gene. *p*-values are shown for European, East Asian, African, and Unknown/Admixed populations. The nominal statistical threshold for *p* = 0.01 is shown. The location of each SNP is shown on the *SLC6A8* gene below the *x*-axis. EUR: European; ASN: East Asian; AFR: African; UNK/MIX: Unknown/Admixed populations.

There were some differences seen in the MAFs calculated for *SLC6A8* variants identified in the ASD patient group compared to those from ethnic populations in 1000 Genomes database (Supplementary Table S4). Figure 6 shows the *p*-values from Fisher’s exact test (plotted as -log (*p*)) and the variants identified. Only three of these differences were significant (Fisher’s exact test, *p* = 0.01), but after running the Benjamini–Hochberg procedure, these values fell below the threshold of significance.

## 3. Discussion

Three rare, non-synonymous genetic variants were identified in coding regions in *GATM*, *GAMT* and *SLC6A8* genes in four individuals with ASD. The variants were heterozygous in *GATM* and *GAMT*, implying possible carrier status for a pathogenic mutation; and hemizygous for *SLC6A8* in a single male, suggesting creatine transporter deficiency if the variant is proven pathogenic. All three variants were predicted as being damaging using SIFT, PolyPhen-2 and MutationTaster. No creatine transporter defect had been demonstrated in males with the hemizygous *SLC6A8* c.1162G > A variant. This variant was subsequently proven to be benign, based on its presence in two patients with mental retardation and no creatine transporter defect [31]. The variants in *GATM* and *GAMT* can only be proven as truly pathogenic if functional studies are performed. No homozygous or compound-heterozygous variants classified as “damaging” were identified in the ASD population, suggesting the prevalence of CDS in autism patients in our sample is zero. We have recently published another study confirming the prevalence to be zero, as no cases of CDS were identified in 443 children with ASD (screening of patients was done using both metabolic and molecular methods) [5]. Schiff et al. [34] screened urine from 203 children with non-syndromic autism and found none to be affected with CDS. The only other genetic screen was carried out by Newmeyer et al [35]. They screened 100 males with ASD for mutations in the *SLC6A8* gene, and reported one affected child. However, this male patient had the c.1162G > A variant. He was later further investigated and had a normal urine creatine/creatinine ratio, suggesting the variant was benign.

### Statistical Differences between MAFs in CDS Genes in ASD and the General Population

Fisher’s exact test was used to determine the statistical significance of any differences identified in the allele frequencies of the autism cohort compared to the 1000 Genomes database (divided into four population groups).

There were some differences seen in the MAFs calculated for *GATM*, *GAMT* and *SLC6A8* variants identified in the ASD patient group, but the only statistically significant results were seen in the *GATM* gene. These included variants in non-coding regions (3′ and 5′UTRs, introns) as well as changes in exons. The Asian autism cohort was compared to the East Asian data in 1000 Genomes initially. Nine variants were identified at a statistically lower frequency in our Asian autism cohort compared to the 1000 Genomes database. The data for these nine variants was then compared to the South Asian data in 1000 Genomes, which further suggested that the MAFs were not significantly different. We do not know the diversity of ethnicities within our Asian cohort and so cannot determine if our population is skewed towards one or another subpopulation. This potentially interesting finding would need further investigation with Asian subpopulations. Two variants in the European autism cohort also presented at a statistically lower allele frequency than in the 1000 Genomes database.

## 4. Materials and Methods

A prospective group consisting of 71 subjects was enrolled in Toronto, and a retrospective sample of 95 subjects was collected as part of the Canadian Autism Genome Project (families with only one autistic child). The former was also part of the prevalence study of CDS in ASD [5]. Supplementary Table S1 shows the cohort divided by ethnicity and sex.

Participants received a diagnosis of ASD from clinician experts based on diagnostic and statistical manual of mental disorders, 4th edition (DSM-IV) criteria for ASD (autism, Asperger’s, or pervasive developmental disorder—not otherwise specified (PDD-NOS)) with the diagnosis confirmed by the autism diagnostic observation schedule (ADOS-G) [36]. An additional group of children with moderate to severe ASD eligible for a publicly-funded Intensive Behavioural Intervention Therapy program for children with ASD was recruited. Children included from this subgroup also underwent assessment by clinician experts, however, rather than the ADOS, their assessment included observation with the Childhood Autism Rating Scale (CARS) [37] and the DSM-IV checklist. Individuals were excluded if they had Rett syndrome, Childhood disintegrative disorder, severe bilateral visual impairment, or severe bilateral hearing impairment.

### 4.1. Study Design and Measurement

To investigate the genetic variability in genes involved in creatine metabolism in children with ASD, Sanger-based DNA sequencing chemistry was used (ABI-3730) to sequence the three genes, *GATM* (AGAT), *GAMT*, and *SLC6A8* (CrT). The entire gene was sequenced including 3′ and 5′UTRs, coding regions and flanking intronic segments. Examination of raw sequence data was completed manually for missense, nonsense, or small insertion/deletion events, by aligning sequences with NCBI reference genes. Genetic variations were compared to sequence data in Alamut (Alamut Visual version 2.6, Interactive Biosoftware, Rouen, France) and three variation databases: the 1000 Genomes phase 3 dataset [38], Exome Sequencing Project [39] and the Exome Aggregation Consortium [40]. Fisher’s exact test was used to calculate “goodness-of-fit” between the allele frequencies in published databases (primarily the 1000 Genomes phase 3 dataset and the autistic patient population, based on ethnicity. If ethnicity was not known, or admixed, then allele frequencies were compared to the combined allele frequency for all ethnicities. Significant variation between allele frequencies for the two populations was noted when *p* < 0.01.

To reduce the false discovery rate, and help reduce type 1 errors (false positives) we applied the Benjamini–Hochberg procedure. The variants for each ethnic population for each gene (excluding the novel variants) were ranked in ascending *p*-values. Each individual *p*-value’s Benjamini–Hochberg critical value was calculated using the formula (i/m)Q (with i = the individual *p*-value’s rank; m = total number of tests; and Q = the false discovery rate (0.01)). The highest *p*-value that was also smaller than the critical value was noted, and that value and all values with lower *p*-values were considered significant. These variants are identified on the Manhattan plots.

### 4.2. Molecular Genetics

DNA was prepared from blood or lymphoblasts; PCR products were amplified using the primers listed in Supplementary Table S5, and sequenced by TCAG (Toronto Center of Applied Genomics, Toronto, ON, Canada). *GATM* gDNA was amplified using Herculase II Fusion Taq polymerase (Agilent Tech., Mississauga, ON, Canada). *GAMT* gDNA was amplified using Hotstar Taq polymerase (Qiagen, Toronto, ON, Canada). In some cases, a second nested amplification was carried out on the first PCR product; some of these primers had a GC clamp at their 5′ end. *SLC6A8* was amplified using Herculase II Fusion Taq polymerase, Hotstar Taq polymerase or TaKaRa La Taq polymerase (Takara Bio Inc., Shiga, Japan). Some of these primers had an M13 clamp at their 5’ end (G. Salomons, personal communication).

### 4.3. Ethics, Consent and Permissions

The study was approved by the Research Ethics Boards of The Hospital of Sick Children, Surrey Place Center, and Holland Bloorview Kids Rehabilitation. Subjects enrolled in the study after parents completed written informed consent.

## 5. Conclusions

We hypothesized that genetic variability (polymorphisms) in the three genes associated with CDS could impact the health of children and result in an autistic phenotype. This hypothesis was suggested by the fact that rare variants have been shown to have a cumulative effect, contributing to ASD risk (narrow sense heritability); and that CSD and ASD have overlapping phenotypes suggesting some similar disease mechanisms.

Our findings suggest there could be a lower association of some specific *GATM* gene variants in Asians and Europeans with autism compared to the 1000 Genomes database (East Asian population and all Europeans), observations that would need to be corroborated in a larger group of autism patients in which ethnic sub-populations are known. Variants in *GATM* have not been associated with autism previously, nor has the chromosomal region of 15q21.1. There are no genes overlapping *GATM* that have been associated with autism.

By sequencing the three CSD genes, the findings demonstrate that genetic variability in genes of biosynthesis (AGAT and GAMT) and transport of creatine (CrT) is unlikely to play a part in the pathogenesis of ASD in children. Through this work, we have corroborated results determined in our prior prevalence study in which none of the 443 children with ASD was found to be affected with CDS [5]. Both studies taken together suggest that, despite the possibility that there is a shared feature that might cause the separate but similar ASD and CDS autistic symptoms, we should look elsewhere for causation of ASD.

## Figures and Tables

**Table 1:** Summary of *GATM* (glycine amidinotransferase) gene sequencing results. Variants identified in the autisim spectrum disorder (ASD) population are noted, and minor allele frequency (MAF) calculated. MAF of variants present in 1000 Genomes, Exome Sequencing Project (ESP) and Exome Aggregation Consortium (ExAC) databases are shown for comparison. ^1^ Total number of samples = 166 (332 alleles); ^2^ MAF = minor allele frequency; the number of alleles in which variant was found/total number of alleles; ^3^ MAC = minor allele count; the number of times the minor allele was observed in the sample population of chromosomes; ^4^ Polymorphic variant: (>0.01 MAF in one database); ^5^ Rare variant: ≤0.01 MAF in at least one published database; ^6^ Novel variant: Not present in any published database; coding sequence (exons) are in bold. SNP: single nucleotide polymorphism; UTR: untranslated region; and SIFT: ‘sorting tolerant from intolerant’ algorithm.

			Autism Population (*n* = 166, Alleles = 332 ^1^)			Databases			
*GATM* Exon/Intron	DNA Change/Protein Change	SNP ID	Homozygous/Heterozygous Change ^1^	Number of Observed Alleles	MAF ^2^	1000 Genomes (Phase 3) MAF, MAC ^3^)	ESP Report (July 2013) MAF, MAC	ExAC (January 2015) MAF, MAC	Comments
5UTR	c. − 200C > T	rs7164139	Homo: 20	118	0.355	0.444	x	0.5533	polymorphic variant ^4^
			Hetero: 78			2223/5008		83/150	
5UTR	c. − 140C > T	rs533626184	Hetero: 1	1	0.003	0.0022	x	x	rare variant
						11/5008			
5UTR	c. − 30T > G	rs8024550	Hetero: 5	5	0.015	0.144	0.073	0.07742	polymorphic variant
						720/5008	651/8926	155/2002	
intron 1	c.70 − 77C > T	rs12437887	Homo: 20	116	0.349	0.443	x	x	polymorphic variant
			Hetero: 76			2221/5008			
intron 1	c.70 − 38G > T	rs12437840	Homo: 23	119	0.358	0.443	0.270	0.3827	polymorphic variant
			Hetero: 73			2219/5008	3509/12992	24153/63112	
**exon 2**	**c.282G > A**	**rs141223762**	**Hetero: 1**	**1**	**0.003**	**NA**	**0.000154**	**0.0001153**	**rare variant ^5^**
	**p.=**						**2/12992**	**14/121390**	
intron 2	c.289 − 76T > C	rs540536879	Hetero: 3	3	0.009	0.000998	x	x	rare variant
						5/5008			
intron 2	c.289 − 34T > C	rs74009633	Hetero: 1	1	0.003	0.0096	0.005	0.001947	rare variant
						48/5008	65/12982	193/99144	
intron 2	c.289 − 24G > A	rs145644806	Hetero: 1	1	0.003	0.0002	0.000154	x	rare variant
						1/5008	2/12988		
**exon 3**	**c.330A > T**	**rs1288775**	**Homo: 29**	**132**	**0.398**	**0.619**	**0.435**	**0.581**	**polymorphic variant**
	**p.Q110H**		**Hetero: 74**			**3098/5008**	**5651/12992**	**70416/121104**	
intron 3	c.485-11_dupT		Homo: 1	2	0.006	x	x	x	novel variant ^6^
**exon 5**	**c.700G > C**		**Hetero: 1**	**1**	**0.003**	**NA**	**x**	**0.00001651**	**rare variant**
	**p.D234H**							**2/121104**	**SIFT: deleterious (score: 0); PolyPhen-2: probably damaging (score: 0.986); MutationTaster: disease-causing (*p*-value: 1.0)**
intron 5	c.813 + 46C > G	rs150282769	Hetero: 1	1	0.003	0.0002	0.000847	0.0004	rare variant
						1/5008	11/12992	46/118404	
intron 6	c.978 + 43T > C		Hetero: 1	1	0.003	x	x	x	novel variant
intron 6	c.979 − 316A > G	rs9972405	Homo: 1	40	0.120	0.0998	x	x	polymorphic variant
			Hetero: 38			500/5008			
intron 6	c.979-49_979-51delTAA	rs200176845	Homo: 1	2	0.006	0.0100	0.0137	0.00228	rare variant
						50/5008	171/12480	248/108652	
intron 6	c.979 − 39T > C		Hetero: 1	1	0.003	x	x	x	novel variant
intron 7	c.1043 − 254A > G	rs57369693	Homo: 1	40	0.120	0.0998	x	x	polymorphic variant
			Hetero: 38			500/5008			
intron 8	c.1159 + 39A > G	rs113129788	Hetero: 1	1	0.003	0.0002	x	0.000019	rare variant
						1/5008		2/105236	
intron 8	c.1160 − 46C > T	rs201589362	Hetero: 2	2	0.006	0.0002		0.00006987	rare variant
						1/5008		7/100180	
**exon 9**	**c.1252T > C**	**rs1145086**	**Homo: 46**	**172**	**0.518**	**0.2823**	**0.534**	**0.5329**	**polymorphic variant**
	**p.=**		**Hetero: 80**			**1414/5008**	**6937/12992**	**64562/121146**	
3UTR	c.*27C > G	rs200143728	Hetero: 1	1	0.003	NA	x	0.0004716	rare variant
								56/118754	
3UTR	c.*125G > A	rs143689218	Hetero: 2	2	0.006	0.0086	x	x	rare variant
						43/5008			
3UTR	c.*411G > A		Hetero: 1	1	0.003	x	x	x	novel variant
3UTR	c.*600A > G	rs1049503	Homo: 23	120	0.361	0.4507	x	x	polymorphic variant
			Hetero: 74			2257/5008			
3UTR	c.*715T > C	rs1049508	Homo: 27	123	0.370	0.618	x	x	polymorphic variant
			Hetero: 69			3094/5008			
3UTR	c.*734_*735insCA	rs35410548	Homo: 46	171	0.515	0.718	x	x	polymorphic variant
			Hetero: 79			3594/5008			
3UTR	c.*913G > A	rs17618637	Hetero: 10	10	0.030	0.0553	x	x	polymorphic variant
						277/5008			
3UTR	c.*940C > T	rs1049518	Homo: 54	108	0.325	0.718	x	x	polymorphic variant
						3594/5008			

**Table 2:** Summary of guanidinoacetate methyltransferase (*GAMT*) gene sequencing results. Variants identified in the ASD population are noted, and minor allele frequency (MAF) calculated. MAF of variants present in 1000 Genomes, ESP and Exome Aggregation Consortium (ExAC) databases are shown for comparison. ^1^ Total number of samples = 166 (332 alleles); ^2^ MAF = minor allele frequency; the number of alleles in which variant was found/total number of alleles; ^3^ MAC = minor allele count; the number of times the minor allele was observed in the sample population of chromosomes; ^4^ Polymorphic variant: (>0.01 MAF in one database); ^5^ Rare variant: ≤0.01 MAF in at least one published database; coding sequence (exons) are in bold; and dbSNP: Single Nucleotide Polymorphism database.

			Autism Population (*n* = 166, Alleles = 332 ^1^)			Databases			
*GAMT* Exon/Intron	DNA Change/Protein Change	SNP ID	Homozygous/Heterozygous Change ^1^	Number of Observed Alleles	MAF ^2^	1000 Genomes (Phase 3) MAF, MAC ^3^	ESP Report (July 2013) MAF, MAC	ExAC (January 2015) MAF, MAC	Comments
intron 1	c.182 − 173G > A	rs112975707	Hetero: 6	6	0.018	0.0491	x	x	polymorphic variant ^4^
						246/5008			
**exon 2**	**c.227C > T**	**rs150338273**	**Hetero: 2**	**2**	**0.006**	**NA**	**0.000616**	**0.000411**	**rare variant ^5^**
	**p.S76L**						**8/12984**	**29/70554**	**SIFT: deleterious (score: 0.03); PolyPhen-2: possibly damaging (score: 0.66); MutationTaster: disease-causing (*p*-value: 0.986)**
intron 2	c.327 + 69T > G	rs266808	Hetero: 4	4	0.012	0.0451	x	x	polymorphic variant
						226/5008			
**exon 3**	**c.348G > A**	**rs117884619**	**Hetero: 1**	**1**	**0.003**	**0.0104**	**x**	**0.003969**	**rare variant, benign allele in dbSNP**
	**p.=**					**52/5008**		**431/108600**	
intron 3	c.391 + 47A > G	rs73515058	Hetero: 12	12	0.036	0.0865	0.0852	0.06853	polymorphic variant
						433/5008	1106/12988	5944/86734	
intron 4	c.460 − 31G > A	rs55776826	Homo: 2	45	0.136	0.1132	0.153	0.1294	polymorphic variant
			Hetero: 41			567/5008	1989/13006	15585/120422	
intron 5	c.571 − 60C > T	rs266809	Homo: 1	8	0.024	0.0731			polymorphic variant
			Hetero: 6			366/5008			
intron 5	c.571 − 6G > A	rs2074899	Hetero: 22	22	0.066	0.1088	0.02	0.06984	polymorphic variant
						545/5008	265/13000	8254/118186	
**exon 6**	**c.626C > T**	**rs17851582**	**Hetero: 23**	**23**	**0.069**	**0.0365**	**0.071**	**0.07554**	**polymorphic variant**
	**p.T209M**					**183/5008**	**926/13004**	**8958/118584**	
3UTR	c.*146A > G	rs659455	Homo: 1	7	0.021	0.0765	x	x	polymorphic variant
			Hetero: 5			383/5008			
3UTR	c.*151T > C	rs659460	Homo: 1	7	0.021	0.0761	x	x	polymorphic variant
			Hetero: 5			381/5008			
3UTR	c.*276C > T	rs266810	Homo: 1	7	0.021	0.0761	x	x	polymorphic variant
			Hetero: 5			381/5008			
3UTR	c.*311C > G	rs266811	Homo: 1	7	0.021	0.0761	x	x	polymorphic variant
			Hetero: 5			381/5008			
3UTR	c.*369C > A	rs75762821	Hetero: 3	3	0.009	0.006	x	x	rare variant
						28/5008			
3UTR	c.*388C > T	rs266812	Homo: 1	7	0.021	0.0757	x	x	polymorphic variant
			Hetero: 5			379/5008			
3UTR	c.*406A > G	rs266813	Homo: 2	19	0.057	0.274	x	x	polymorphic variant
			Hetero: 15			1372/5008			

**Table 3:** Summary of solute carrier family 6 member 8 (*SLC6A8*) gene sequencing results. Variants identified in the ASD population are noted, and minor allele frequency (MAF) calculated. MAF of variants present in 1000 Genomes, ESP and Exome Aggregation Consortium ExAC databases are shown for comparison. ^1^ Total number of samples = 166 (134 males, 32 females, 198 alleles); ^2^ MAF = minor allele frequency; the number of alleles in which variant was found/total number of alleles; ^3^ MAC = minor allele count; the number of times the minor allele was observed in the sample population of chromosomes; ^4^ Polymorphic variant: (>0.01 MAF in one database); ^5^ Rare variant: ≤0.01 MAF in at least one published database; and ^6^ Novel variant: Not present in any published database; coding sequence (exons) are in bold.

			Autism Pop (*n* = 166, Alleles = 198 ^1^)			Databases			
*SLC6A8* Exon/Intron	DNA Change/Protein Change	SNP ID	Homozygous/Heterozygous Change ^1^	No. Observed Alleles	MAF ^2^	1000 Genomes (Phase 3) MAF, MAC ^3^	ESP Report (July 2013) MAF, MAC	ExAC (January 2015) MAF, MAC	Comments
5UTR	c. − 5 A > G	rs384573	Homo: 166	196	1.000	NA	x	1	polymorphic variant ^4^
								6172/6172	
intron 1	c.262 + 26T > C	rs192387453	Homo: 25M	31	0.158	0.151	0.121	0.1084	polymorphic variant
			Hetero: 6F			570/3775	1249/10352	5029/46406	
intron 1	c.263 − 95G > A		Homo: 1M	1	0.005	x	x	x	novel variant ^6^
intron 2	c.394 + 88G > A	rs6643763	Homo: 21M	28	0.143	0.102	x	x	polymorphic variant
			Hetero: 7F			385/3775			
intron 2	c.394 + 108G > A		Homo: 1M	1	0.005	x	x	x	novel variant
**exon 5**	**c.813C > T**	**rs138064933**	**Homo: 1M**	**1**	**0.005**	**0.001**	**0.0027**	**0.003346**	**rare variant ^5^**
	**p.=**					**2/3775**	**29/10561**	**280/83683**	
intron 5	c.913 − 40T > C	rs187505163	Homo: 1M	1	0.005	0.009	0.014	0.003969	rare variant
						34/3775	148/10563	339/85417	
intron 6	c.1016 + 41dupTGCCC	rs371888321	Homo: 1M	1	0.005	x	x	x	novel variant
intron 6	c.1016 + 56del		Homo: 1M	1	0.005	x	x	x	novel variant
	TGCCC								
intron 7	c.1141 + 18G > A	rs187400676	Hetero: 1F	1	0.005	0.006	0.000284	0.004194	rare variant
						22/3775	3/10563	365/87034	
intron 7	c.1141 + 37G > A	rs2071028	Homo: 20M	24	0.122	0.153	0.127	0.1014	polymorphic variant
			Hetero: 4F			576/3775	1341/10563	8811/86854	
intron 7	c.1141 + 87A > G	rs41302172	Homo: 25 (21M + 4F)	32	0.163	0.101	x	x	polymorphic variant
			Hetero: 3F			383/3775			
intron 7	c.1142 − 130C > T	rs141015652	Homo: 3M	3	0.015	0.021	x	x	polymorphic variant
						81/3775			
intron 7	c.1142 − 69G > A		Homo: 1M	1	0.005	x	x	x	novel variant
intron 7	c.1142 − 35G > A	rs201555047	Homo: 1M	2	0.010	0.006	0.00396	0.008994	rare variant
			Hetero: 1F			21/3775	39/9845	117/13009	
**exon 8**	**c.1162G > A**	**rs374163604**	**Homo: 1M**	**1**	**0.005**	**0.0003**	**0.0002886**	**0.00007051**	**rare variant**
	**p.A388T**					**1/3775**	**3/10394**	**1/14182**	**SIFT: deleterious (score: 0.02); PolyPhen-2: probably damaging (score: 0.969); MutationTaster: disease-causing (*p*-value: 1.0)**
intron 8	c.1255 − 44delG	rs34035058	Homo: 1M	1	0.005	NA	0.000688	0.0002874	rare variant
							7/10180	18/62628	
intron 8	c.1255 − 31C > T	rs193175235	Homo: 2M	2	0.010	0.011	0.00559	0.004952	rare variant
						42/3775	59/10560	378/76334	
intron 9	c.1392 + 31T > C	rs183780161	Homo: 1M	1	0.005	0.003	0.00578	0.001411	rare variant
						10/3775	61/10556	123/87147	
intron 10	c.1495 + 38C > T	rs200729826	Homo: 2M	2	0.010	0.021	0.0000947	0.00997	rare variant
						78/3775	1/10562	858/86058	
intron 10	c.1496 − 17G > A	rs375265267	Homo: 1M	1	0.005	NA	0.000189	0.0000347	rare variant
							2/10563	3/86591	
intron 10	c.1496 − 8C > T	rs376038235	Hetero: 1F	1	0.005	0.002	x	0.001025	rare variant
						9/3775		89/86861	
intron 11	c.1596 + 21G > A	rs73633747	Homo: 1M	1	0.005	0.019	0.0186	0.005616	rare variant
						70/3775	194/10422	445/79237	
intron 12	c.1768 − 3C > T	rs150207268	Hetero: 1F	1	0.005	0.002	0.00398	0.006573	rare variant
						9/3775	42/10554	254/38642	
3UTR	c.*207G > C	rs6571290	Homo: 21M	28	0.143	0.194	x	x	polymorphic variant
			Hetero: 7F			731/3775			

**Table 4:** Summary of glycine amidinotransferase (*GATM*) gene variants that showed significance when compared to East Asian population, with additional data from 1000 Genomes South Asian population. ^1^ Total number of autism cohort Asian samples = 17 (*n* = 34 alleles); ^2^ Number of minor alleles seen in population; ^3^ Number of major alleles seen in the population; ^4^ Minor allele frequency; the number of alleles in which variant was found/total number of alleles.

GATM (AGAT) DNA Change/Protein Change	SNP ID	ASN *n* = 34 ^1^			East ASN (1000 Genomes Phase 3) *n* = 1008			Fisher’s Exact Test ASN	South ASN (1000 Genomes Phase 3) *n* = 978			Fisher’s Exact Test ASN
		**Minor ^2^**	**Major ^3^**	**MAF ^4^**	**Minor**	**Major**	**MAF**		**Minor**	**Major**	**MAF**	
c. − 200C > T	rs7164139	15	19	0.441	811	197	0.805	0.000005	351	627	0.359	0.365138
c.70 − 77C > T	rs12437887	16	18	0.471	813	195	0.807	0.00002	351	627	0.359	0.205191
c.70 − 38G > T	rs12437840	16	18	0.471	813	195	0.807	0.00002	351	627	0.359	0.205191
c.330A > T, p.Q110H	rs1288775	17	17	0.500	825	183	0.818	0.000037	451	527	0.461	0.727522
c.1252T > C, p.=	rs1145086	22	12	0.647	906	102	0.899	0.000114	621	357	0.635	1
c.*600A > G	rs1049503	18	16	0.529	813	195	0.807	0.0003	384	594	0.393	0.112623
c.*715T > C	rs1049508	17	17	0.500	825	183	0.818	0.000037	451	527	0.461	0.727522
c.*734_*735insCA	rs35410548	23	11	0.676	906	102	0.899	0.000499	621	357	0.635	0.718426
c.*940C > T	rs1049518	18	16	0.529	906	102	0.899	0	621	357	0.635	0.21126

## Supplementary Materials

Supplementary materials can be found at www.mdpi.com/1422-0067/18/8/1665/s1.

## Abbreviations

ADOS-G	Autism Diagnostic Observation Schedule
AGAT	Arginine:glycine Amidinotransferase
ASD	Autism Spectrum Disorder
bp	Base Pair
CDS	Creatine Deficiency Syndrome
CARS	Childhood Autism Rating Scale
CrT	Creatine Transporter
ExAC	Exome Aggregation Consortium
GAMT	Guanidinoacetate Methyltransferase
MAC	Minor allele Count
MAF	Minor allele Frequency
NHLBI GO ESP	National Heart, Lung and Blood Institute Grand Opportunity Exome Sequencing Project
PDD-NOS	Pervasive Developmental Disorder–Not Otherwise Specified
SNP	Single Nucleotide Polymorphism
UTR	Untranslated Region
