# Supplementary material for: Variability of Creatine Metabolism Genes in Children with Autism Spectrum Disorder
Source: Int J Mol Sci. 2017 Jul 31;18(8):1665. doi: 10.3390/ijms18081665 (PMC5578055; doi:10.3390/ijms18081665)
Supplement: Supplementary file 1 [file ijms-18-01665-s001.zip › Supplementary Tables legends.pdf]

Supplementary Table 1. Summary of *GATM* gene (AGAT) sequencing results divided by population. Variants identified in the ASD population are noted, and minor allele frequency calculated (MAF). MAF of variants present in 1000 Genomes, are shown. [1]Total number of samples = 166 (n=332 alleles); [2]Number of minor alleles seen in population; [3]Number of major alleles seen in population; [4]Minor allele frequency; the number of alleles in which variant was found/ total number of alleles; *Compared to ExAC database.

Supplementary Table 2. Summary of *GAMT* gene sequencing results divided by population. Variants identified in the ASD population are noted, and minor allele frequency calculated (MAF). MAF of variants present in 1000 Genomes, are shown. [1]Total number of samples = 166 (n=332 alleles); [2]Number of minor alleles seen in population; [3]Number of major alleles seen in population; [4]Minor allele frequency; the number of alleles in which variant was found/ total number of alleles; *Compared to ExAC database.

Supplementary Table 3. Summary of *SLC6A8* gene sequencing results divided by population. Variants identified in the ASD population are noted, and minor allele frequency calculated (MAF). MAF of variants present in 1000 Genomes, are shown. Total number of samples = 166 (32 females, 134 males; n=198 alleles); [2]Number of minor alleles seen in population; [3]Number of major alleles seen in population; [4]Minor allele frequency; the number of alleles in which variant was found/ total number of alleles; *Compared to ExAC database.

Supplementary Table 4. Primers used for amplification of *GATM*, *GAMT* and *SLC6A8* gDNA for sequencing. The GC clamp added to some primers is underlined and M13 clamps are in bold.

Supplementary Table 5. Autism patients enrolled in study, divided by ethnic population and sex (166 patients).
